# Non-Recessive Bt Toxin Resistance Conferred by an Intracellular Cadherin Mutation in Field-Selected Populations of Cotton Bollworm

**DOI:** 10.1371/journal.pone.0053418

**Published:** 2012-12-28

**Authors:** Haonan Zhang, Shuwen Wu, Yihua Yang, Bruce E. Tabashnik, Yidong Wu

**Affiliations:** 1 Key Laboratory of Integrated Management of Crop Diseases and Pests (Ministry of Education), College of Plant Protection, Nanjing Agricultural University, Nanjing, China; 2 Department of Entomology, University of Arizona, Tucson, Arizona, United States of America; University of Tennessee, United States of America

## Abstract

Transgenic crops producing *Bacillus thuringiensis* (Bt) toxins have been planted widely to control insect pests, yet evolution of resistance by the pests can reduce the benefits of this approach. Recessive mutations in the extracellular domain of toxin-binding cadherin proteins that confer resistance to Bt toxin Cry1Ac by disrupting toxin binding have been reported previously in three major lepidopteran pests, including the cotton bollworm, *Helicoverpa armigera*. Here we report a novel allele from cotton bollworm with a deletion in the intracellular domain of cadherin that is genetically linked with non-recessive resistance to Cry1Ac. We discovered this allele in each of three field-selected populations we screened from northern China where Bt cotton producing Cry1Ac has been grown intensively. We expressed four types of cadherin alleles in heterologous cell cultures: susceptible, resistant with the intracellular domain mutation, and two complementary chimeric alleles with and without the mutation. Cells transfected with each of the four cadherin alleles bound Cry1Ac and were killed by Cry1Ac. However, relative to cells transfected with either the susceptible allele or the chimeric allele lacking the intracellular domain mutation, cells transfected with the resistant allele or the chimeric allele containing the intracellular domain mutation were less susceptible to Cry1Ac. These results suggest that the intracellular domain of cadherin is involved in post-binding events that affect toxicity of Cry1Ac. This evidence is consistent with the vital role of the intracellular region of cadherin proposed by the cell signaling model of the mode of action of Bt toxins. Considered together with previously reported data, the results suggest that both pore formation and cell signaling pathways contribute to the efficacy of Bt toxins.

## Introduction

The insecticidal proteins of *Bacillus thuringiensis* (Bt) kill some major insect pests, but are harmless to vertebrates and most other organisms [1], [2]. Transgenic crops producing Bt toxins grew on more than 66 million hectares worldwide in 2011 [3]. The most widely used Bt proteins are crystalline Cry1A toxins, particularly Cry1Ab in Bt corn and Cry1Ac in Bt cotton, which kill lepidopteran larvae [4]. The primary threat to the long-term efficacy of Bt toxins is evolution of resistance by pests [5]–[8]. Some degree of field-evolved resistance to Bt toxins, which entails a genetically based decrease in susceptibility, has been reported in two species exposed to Bt sprays [9], [10] and at least seven species exposed to Bt crops [4], [11]–[18]

**Figure 1:**
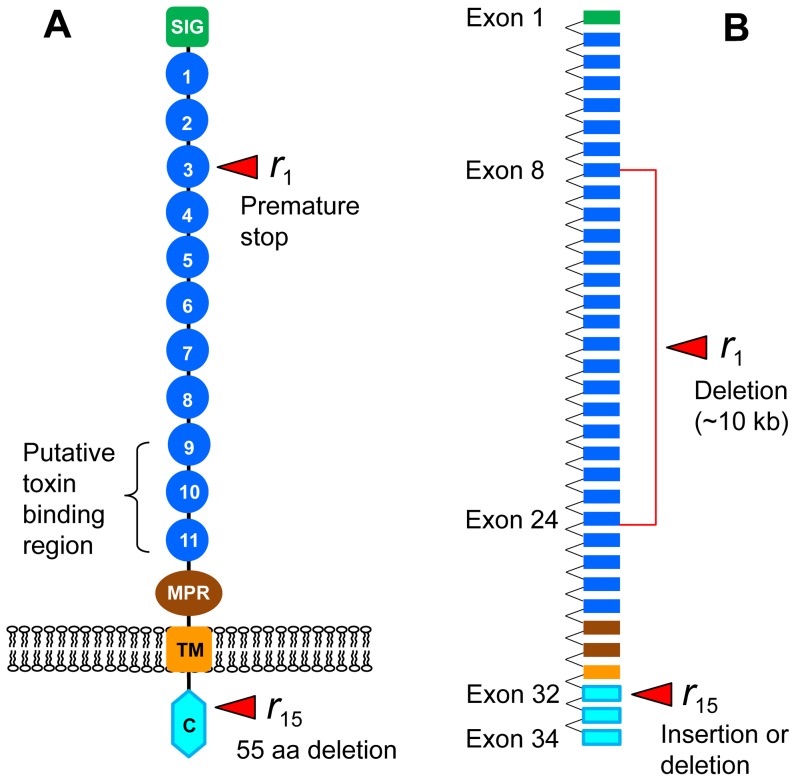
Cadherin protein of *H. armigera* encoded by *HaCad*. **A.** Protein structure of HaCad predicted from cDNA with extracellular region (amino-terminal signal sequence [SIG], 11 cadherin repeats [1]–[11], membrane proximal region [MPR]), transmembrane region (TM), and cytoplasmic domain (C). **B.** Genomic DNA sequence of *HaCad*. Resistance allele *r_1_* has a stop codon at 428G in cadherin repeat 3 caused by a genomic DNA deletion of ca. 10 kb [36]. HaCad encoded by resistance allele *r*_15_ lacks 55 amino acids in the cytoplasmic domain caused by a 165 bp deletion in exon 32. We found three genomic DNA variants of *r*_15_ that cause loss of exon 32, one from each of three field populations: 1459 bp insertion from Xiajin, 92 bp deletion from Anyang, and >5000 bp insertion from Anci.

Cry1A toxins bind to the extracellular domain of cadherin proteins that traverse the larval midgut membrane; disruption of this binding can cause resistance [19]–[22]. Cadherins that bind Bt toxins also have a cytoplasmic domain (Figure 1) that has not been implicated previously in resistance [23]–[26]. The putative importance of the cytoplasmic domain differs between the two leading models of the mode of action of Bt toxins: the pore formation model and the cell signaling model [19], [27]. In the pore formation model, binding of toxin monomers to cadherin promotes generation of toxin oligomers that bind with increased affinity to membrane-bound aminopeptidases N and alkaline phosphatases, subsequently creating pores in the midgut membrane that cause osmotic shock and cell death [19], [28]. By contrast, the cell signaling model proposes that binding of toxin monomers to cadherin activates an intracellular magnesium-dependent signaling pathway that causes cell death [27]. Thus, the cytoplasmic domain of cadherin is essential in the intracellular pathway of the cell signaling model, but has no explicit role in the pore formation model.

Although data are limited on the genetic basis of field-evolved resistance to Bt crops [13], [14], [29], extracellular domain mutations disrupting a cadherin protein that binds Cry1Ac in the larval midgut are tightly linked with recessive resistance to Cry1Ac in some laboratory-selected strains of three major pests targeted by Bt cotton: *Heliothis virescens*, *Pectinophora gossypiella*, and cotton bollworm, *Helicoverpa armigera* [20]–[22]. Non-recessive resistance to Cry1Ac has also been seen in both laboratory- and field-selected strains of *H. armigera* [29]–[33], but the mutations causing non-recessive resistance to Bt toxins have not been characterized before for any insect. Dominance can be quantified with the parameter *h*, which varies from 0 for completely recessive resistance to 1 for completely dominant resistance [34]. Because the term “dominant” sometimes implies complete dominance, we use “non-recessive” to denote resistance that differs substantially from completely recessive resistance [29].

Here we discovered a novel cadherin allele genetically linked with non-recessive resistance of *H. armigera* to Cry1Ac. We found this allele in three field-selected populations, each from a different province in northern China where Bt cotton that produces Cry1Ac has been grown intensively for many years [17]. Whereas previously characterized recessive cadherin mutations linked with resistance to Cry1Ac occur in the extracellular region of cadherin [20]–[22], the non-recessive mutation identified here occurs in the cytoplasmic domain of cadherin. These results suggest that the cytoplasmic domain of cadherin contributes to toxicity of Cry1Ac.

## Results

### Identification of a cadherin allele (*r*_15_) with a cytoplasmic domain deletion

We discovered a novel cadherin resistance allele while screening *H. armigera* collected in 2009 from three field populations in northern China that had been exposed intensively to Bt cotton. To detect resistance alleles, we used an F_1_ screen of 572 single-pair families obtained by crossing moths derived from each of the three field populations with moths from the laboratory-selected resistant SCD-r1 strain (Table S1). The SCD-r1 strain was homozygous for the *r*_1_ cadherin allele (*r*_1_ *r*_1_) that carries a premature stop codon in the extracellular domain of *HaCad* [35]. We sequenced the cadherin cDNA of F_1_ progeny that survived exposure to a diagnostic concentration of Cry1Ac (1 µg Cry1Ac per cm^2^ diet) from 48 of the 112 single-pair families in which resistance was detected (Table S1).

Sequencing of cDNA revealed that 3 of the 48 resistant F_1_ families had a 165 bp in-frame deletion in exon 32 of the cytoplasmic domain of cadherin (Figure 1). The missing 165 bp of exon 32 encode 55 amino acids near the 5′-end of the cytoplasmic domain. Following the nomenclature for resistance alleles of *HaCad* (*r*_1_-*r*_14_) [29], [36]–[38], we name this *HaCad* resistance allele *r*_15_. We detected one *r*_15_ allele in each of the three field populations screened: Xiajin (Shandong Province), Anyang (Henan Province), and Anci (Hebei Province) (GenBank nos. JN898956, JX233819, and JX233820, respectively). For these three populations pooled, the estimated *r*_15_ allele frequency is 0.0061 (95% confidence interval = 0.0016–0.019) (Table S1).

Although the *r*_15_ allele from each population has the same deletion in its cDNA sequence caused by loss of exon 32, the genomic DNA sequence in exon 32 varies among the three populations, indicating at least three independent origins for this allele (Figure S1). The *r*_15_ allele from Xiajin has a 1459 bp insertion (GenBank no. JN898957) with 95% identity to part of the 3′ non-coding sequence of a carboxyl/choline esterase gene of *H. armigera* (GenBank no. FJ997310.1). The *r*_15_ allele from Anyang has a 92 bp deletion in exon 32 (Figure S1). In contrast with the *r*_15_ alleles from Xiajin and Anyang, we obtained no products when we attempted to use high-fidelity PCR to amplify the genomic DNA flanking the deletion in the *r*_15_ allele from Anci. These results suggest that insertion of a DNA fragment >5 kb near exon 32 caused the loss of this exon in the *r*_15_ allele from Anci. To further analyze the resistance conferred by *r*_15_, we established two resistant strains homozygous for the *r*_15_ allele (*r*_15_ *r*_15_), one from Xiajin (XJ-r15) and the other from Anyang (AY-r15) (Figure S2).

### Dominance, magnitude of resistance, and cross-resistance associated with *r*_15_

**Figure 2:**
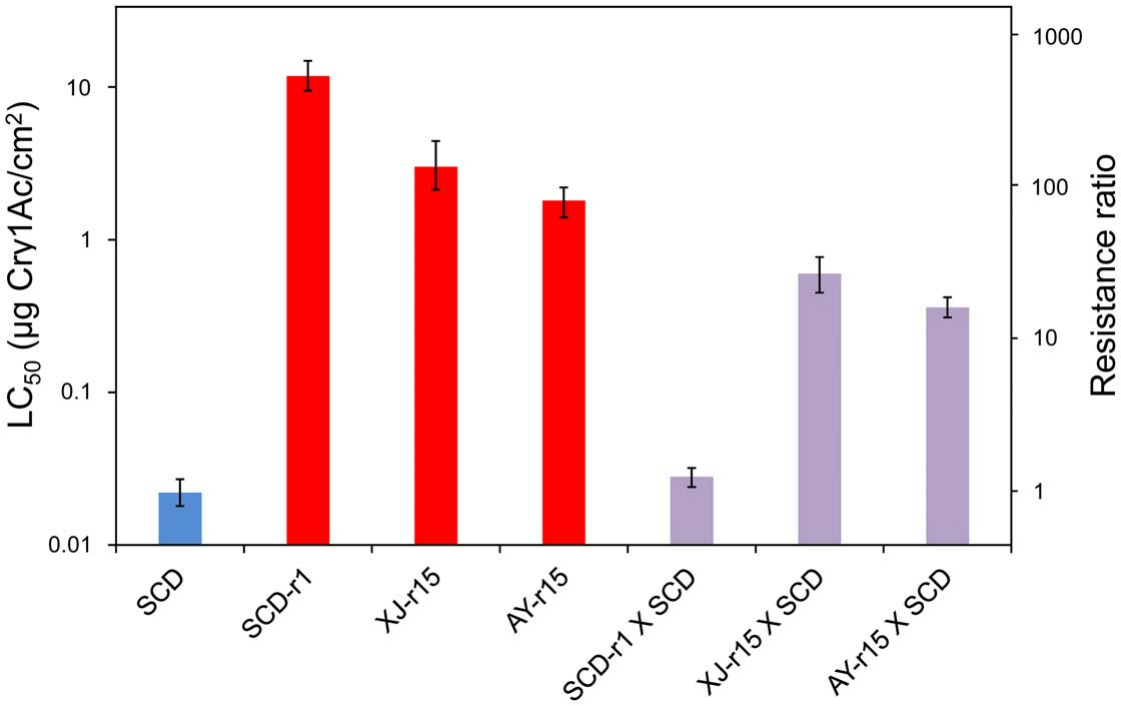
Responses to Bt toxin Cry1Ac of *H. armigera* from a susceptible strain (SCD, blue), three resistant strains (red), and the F_1_ progeny from crosses between each resistant strain and the susceptible strain (purple). SCD-r1: resistant strain with allele *r_1_* affecting the extracellular domain of HaCad. XJ-r15 and AY-r15: resistant strains (from Xiajin and Anyang, respectively) with allele *r*_15_ affecting the cytoplasmic domain of HaCad. Resistance ratio is the concentration killing 50% of larvae (LC_50_) of each strain or group of F_1_ progeny divided by the LC_50_ for the susceptible SCD strain. The black bars show the 95% fiducial limits for LC_50_.

Responses to Cry1Ac of the F_1_ progeny of crosses between the susceptible strain SCD and either XJ-r15 or AY-r15 indicate that the resistance associated with *r*_15_ is not recessive (Figure 2, mean *h* = 0.57, range = 0.32 to 0.70, Table 1). In contrast, consistent with previous work [22], [35], resistance to Cry1Ac was recessive in the SCD-r1 strain (*h* = 0.00 to 0.04, Table 1), which is homozygous for the *r*_1_ allele. The F_1_ progeny from each of the two reciprocal crosses between susceptible strain SCD and either XJ-r15 or AY-r15 responded similarly to Cry1Ac (Table 1). This indicates autosomal inheritance, consistent with previous analyses of *HaCad* [22], [35].

**Table 1:** Magnitude and dominance of resistance to Cry1Ac in *H. armigera* associated with cadherin resistance alleles: *r*_15_ in the cytoplasmic domain and *r*_1_ in the extracellular region.

Source	LC_50_ (95% FL)^a^	Slope ± SE	n	RR^b^	Surv. (%)^c^	Dominance (*h*)^d^	
						LC_50_ e	Surv.^f^
*Strain*							
XJ-r15 (resistant)	3.0 (2.1–4.5)	1.1±0.2	240	140	62	-g	-
AY-r15 (resistant)	1.8 (1.4–2.2)	1.6±0.1	1008	82	60	-	-
SCD-r1 (resistant)	12 (9.5–15)	1.9±0.2	240	540	96	-	-
SCD (susceptible)	0.022 (0.018–0.027)	1.6±0.1	1488	1.0	0	-	-
*Resistant strain*×*susceptible strain*							
XJ-r15*♂*×SCD♀	0.52 (0.23–0.94)	1.9±0.3	240	24	40	0.64	0.65
XJ-r15♀×SCD*♂*	0.69 (0.48–0.95)	2.7±0.4	288	31	40	0.70	0.65
XJ-r15×SCD^h^	0.60 (0.45–0.75)	2.2±0.2	528	27	40	0.68	0.65
AY-r15*♂*×SCD♀	0.38 (0.32–0.47)	2.3±0.3	288	17	21	0.65	0.35
AY-r15♀×SCD*♂*	0.33 (0.26–0.43)	1.8±0.2	288	15	19	0.61	0.32
AY-r15×SCD^h^	0.36 (0.31–0.42)	2.0±0.2	576	16	20	0.63	0.33
SCD-r1×SCD^h^	0.028 (0.024–0.032)	1.9±0.1	864	1.3	0	0.04	0.00
*Resistant strain* (*r*_15_)×*resistant strain* (*r*_1_)							
XJ-r15♀×SCD-r1*♂*	20 (12–47)	1.1±0.5	240	910	100	-	-
AY-r15♀×SCD-r1*♂*	14 (9.0–23)	1.1±0.2	288	640	92	-	-

a Concentration killing 50% of larvae and 95% fiducial limits (µg Cry1Ac per cm^2^ diet).

b Resistance ratio = LC_50_ of a strain or F_1_ progeny from a cross divided by LC_50_ of the susceptible SCD strain.

c Survival at the diagnostic concentration (1 µg Cry1Ac per cm^2^ diet), n = 48.

d *h* ranges from 0 for completely susceptible to 1 for completely dominant.

e *h* calculated from LC_50_ values [34].

f *h* calculated from survival at the diagnostic concentration [34].

g *h* is calculated only for F_1_ progeny from crosses between resistant and susceptible strains.

h Results pooled from the two reciprocal crosses.

We calculated resistance ratios based on the concentration of toxin killing 50% of larvae (LC_50_) of a strain divided by the LC_50_ of the susceptible SCD strain. Resistance ratios for Cry1Ac were 140 for XJ-r15, 82 for AY-r15, and 540 for SCD-r1 (Table 1, Figure 2). Although all three of these strains were highly resistant, the LC_50_ value was significantly higher for SCD-r1 compared with XJ-r15 and AY-r15 (Table 1). Tests of XJ-r15 revealed resistance ratios of 27 for Cry1Aa, 6.3 for Cry1Ab, and 1.4 for Cry2Ab (Table S2), indicating moderate cross-resistance to Cry1Aa and Cry1Ab, but little or no cross-resistance to Cry2Ab.

### Test for allelism and genetic linkage between *r*_15_ and resistance to Cry1Ac

To test for allelism between the *r*_1_ cadherin resistance allele in strain SCD-r1 and any major resistance alleles in strains XJ-r15 and AY-r15, we crossed SCD-r1 with XJ-r15 and AY-r15. This yielded F_1_ progeny with a resistance ratio of 910 for XJ-r15×SCD-r1 and 640 for AY-r15×SCD-r1 (Table 1). Based on both LC_50_ values and survival at the diagnostic concentration of Cry1Ac, the F_1_ progeny from the cross between SCD-r1 and either XJ-r15 or AY-r15 were not less resistant than their parent strains (Table 1). This implies that any major resistance alleles in XJ-r15 and AY-r15 are allelic with *r*_1_ and thus occur at the cadherin locus. This conclusion was confirmed when we calculated the index of commonality (C), which ranges from close to or <0 for resistance conferred by alleles at different loci to close to or >1 for resistance conferred by alleles at a shared locus [29]. The value of C was 1.1 for crosses between SCD-r1 and XJ-r15 and for crosses between SCD-r1 and AY-r15, indicating that the resistance in these three strains was conferred by the same locus.

**Figure 3:**
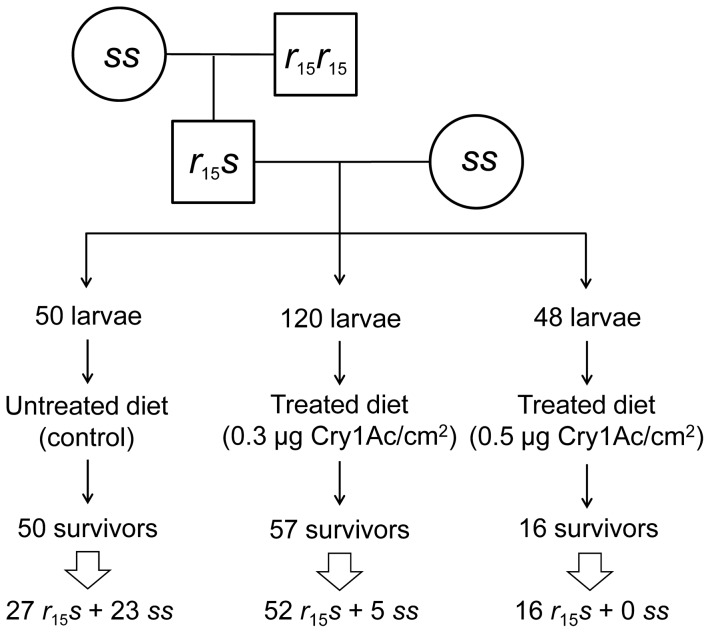
Genetic linkage between the cytoplasmic domain mutant of *HaCad* (*r*_15_) and resistance to Cry1Ac in the XJ-r15 strain of *H. armigera*. We crossed a female (*ss*) from the susceptible SCD strain with a male from the resistant XJ-r15 strain (*r*_15_ *r*_15_) to produce the F_1_ family (*r*_15_ *s*). Next we crossed an F_1_ male (*r*_15_ *s*) with a susceptible SCD female (*ss*) to produce a backcross family from which larvae were placed on untreated diet (control) or diet treated with either 0.3 or 0.5 µg Cry1Ac per cm^2^. After 5 days, all survivors were transferred to untreated diet, reared to the final instar, and genotyped. The frequency of heterozygotes (*r*_15_ *s*) relative to susceptible homozygotes (*ss*) was significantly higher for survivors on treated diet (68∶5) than for survivors on untreated diet (27∶23) (Fisher's exact test, P<0.0001).

Results of a genetic linkage analysis confirm that resistance to Cry1Ac in the XJ-r15 strain is not recessive and is tightly linked with the *r*_15_ allele (Figure 3). In the progeny of a backcross between a male F_1_ (XJ-r15×SCD) and a female SCD, the number of heterozygotes (*r*_15_ *s*) relative to susceptible homozygotes (*ss*) was significantly higher for survivors on treated diet (68 to 5) than on untreated diet (27 to 23) (Fisher's exact test, P<0.0001) (Figure 3). Whereas the ratio of *r*_15_ *s* to *ss* survivors on untreated diet did not differ significantly from the expected 1∶1 ratio (Fisher's exact test, P = 0.84), all 16 survivors on diet treated with the highest toxin concentration used in the linkage analysis (0.5 µg Cry1Ac per cm^2^ diet) were *r*_15_ *s* (Fisher's exact test, P = 0.002) (Figure 3). Because crossing over occurs in male moths and an F_1_ male was a parent for the backcross family [39], the perfect association between survival at this concentration and the *r*_15_ allele indicates tight linkage between resistance to Cry1Ac and the cadherin locus.

### Binding and toxicity of Cry1Ac to Sf9 cells expressing different cadherin alleles

To understand the role of *r*_15_ in the mode of action of Cry1Ac, we expressed four cadherin alleles in Sf9 cells: susceptible (*s*), *r*_15_, and two chimeric alleles (Figure S3). The chimeric allele *s*/*r*_15_ had the cytoplasmic domain from *r*_15_ and the other components from *s*, while the complementary chimeric allele *r*_15_/*s* had the cytoplasmic domain from *s* and the other components from *r*_15_.

**Figure 4:**
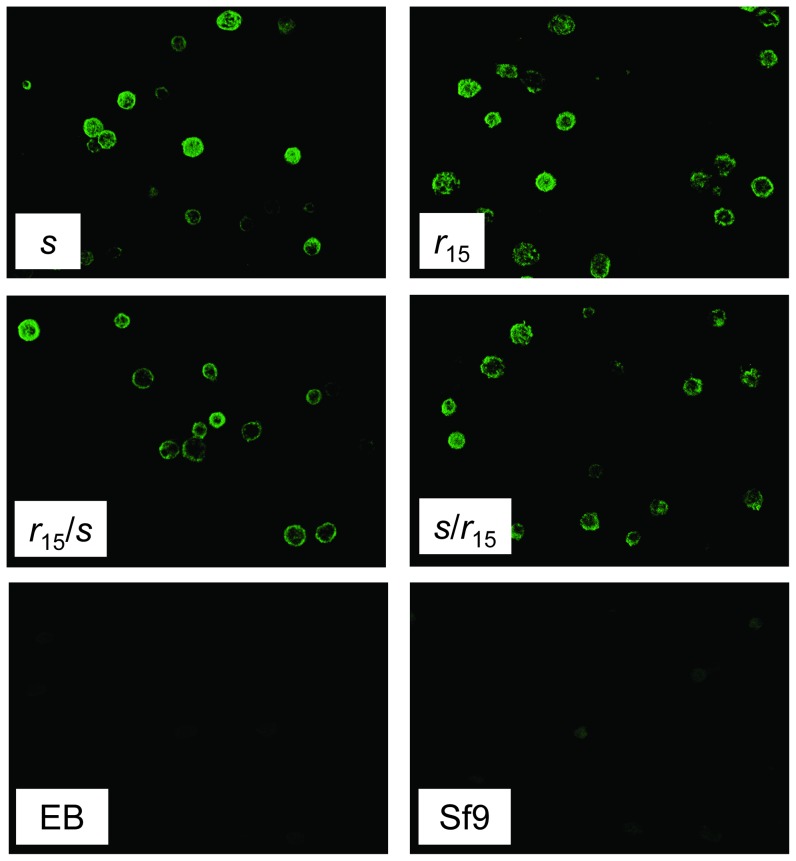
Cry1Ac binding to Sf9 cells transfected with four alleles of *HaCad*. *s*: susceptible allele. *r*_15_: resistant allele, encoding cadherin with a 55 amino acid deletion in the cytoplasmic domain (C). *s*/*r*_15_: chimeric allele with C from *r*_15_ and the other components from *s*. *r*_15_/*s*: complementary chimeric allele with C from *s* and the other components from *r*_15_. Cells were treated with 10 nM Cry1Ac, then probed sequentially with anti-Cry1Ac antiserum (1∶100) and FITC-conjugated anti-rabbit antibody (1∶100). No Cry1Ac binding was detected in control cells that were either transfected with an empty bacmid (EB) or not transfected (Sf9).

Sf9 cells transfected with each of the four cadherin alleles had similar fluorescence signal patterns indicating enriched cadherin expression in their cell membranes and binding of Cry1Ac (Figures 4 and S4). These results show that *r*_15_ did not block cadherin expression on the cell membrane surface or binding of Cry1Ac. As expected, no fluorescence or binding of Cry1Ac was detected in the two control Sf9 cell cultures that were either not transfected or were transfected with an empty bacmid (Figures 4 and S4).

**Figure 5:**
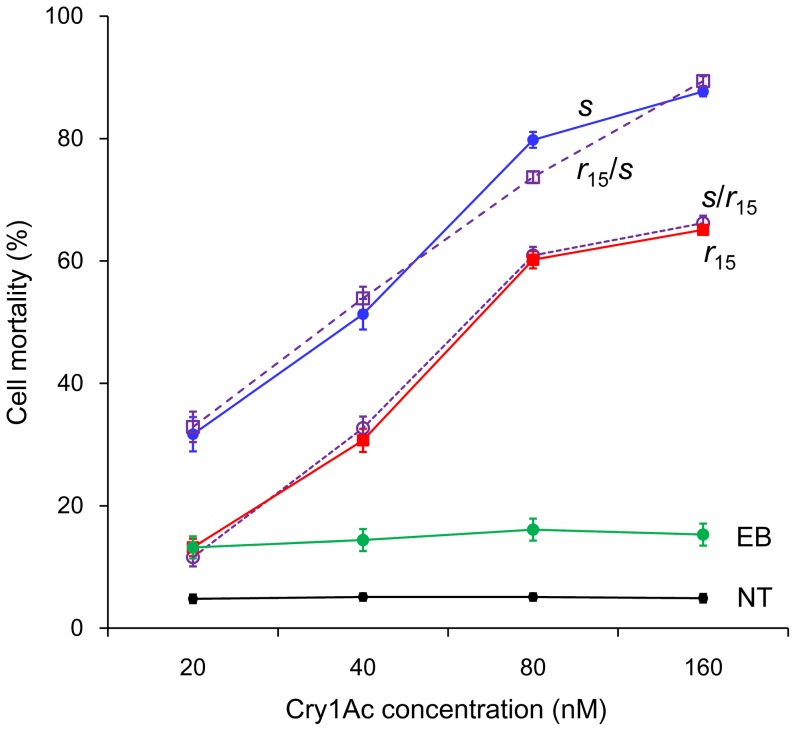
Mortality of Sf9 cells exposed to Cry1Ac. Sf9 cells were transfected with one of four alleles of *HaCad* (*s*, *r*_15_, *r*_15_ */s*, and *s/r*_15_; see Figures 4 and S2 for details) or an empty bacmid (EB), or were not transfected (NT). For cells transfected with alleles of *HaCad*, LC_50_ values (95% FL) were significantly higher for alleles with the cytoplasmic domain of *r*_15_ (*r*_15_: 85 [71–110] and *s/r*_15_: 82 [68–100]) than for alleles with the cytoplasmic domain of *s* (*s*: 38 [31–46] or *r*_15_ */s*: 38 [31–45]). LC_50_ values did not differ significantly between Sf9 cells transfected with alleles of *HaCad* that had the same cytoplasmic domain (*r*_15_ and *s/r*_15_; *s* and *r*_15_ */s*).

As the concentration of Cry1Ac increased, mortality increased for the four Sf9 cell cultures transfected with cadherin alleles, but not for the two control cell cultures (Figure 5). Thus, expression of each of the four cadherin alleles rendered Sf9 cells somewhat sensitive to Cry1Ac. However, relative to Sf9 cells with the cytoplasmic domain encoded by the *s* allele (*s* and *r*_15_/*s*), Sf9 cells with the cytoplasmic domain encoded by the *r*_15_ allele (*r*_15_ and *s*/*r*_15_) were significantly less susceptible to Cry1Ac (Figure 5). Furthermore, susceptibility to Cry1Ac did not differ significantly between cells with the *s* or *r*_15_/*s* allele, or between cells with the *r*_15_ or *s*/*r*_15_ allele (Figure 5). These results show that the cytoplasmic domain affected toxicity of Cry1Ac to Sf9 cells transfected with *HaCad* alleles.

## Discussion

The results here demonstrating that the cytoplasmic domain of cadherin contributes to susceptibility of *H. armigera* to Cry1Ac provide insight into Bt toxin mode of action. Binding of toxin to cadherin is a key step in both the pore formation and cell signaling models, yet the precise role of cadherin in mediating toxicity has been controversial, and the role of the cytoplasmic domain of cadherin is more prominent in the cell signaling model [19], [24], [27], [40]. The results here show that a cytoplasmic domain mutation in cadherin is linked with non-recessive resistance to Cry1Ac. Moreover, although all Sf9 cell cultures we transfected with cadherin alleles bound Cry1Ac, the cytoplasmic domain of the cadherin alleles affected susceptibility to Cry1Ac (Figure 5).

Although the data here do not directly address many of the specific elements of the cell signaling model, they do provide strong evidence that the cytoplasmic domain of cadherin contributes to susceptibility of *H. armigera* to Cry1Ac. Binding of Cry1Ac occurred in cells transfected with either a susceptible cadherin allele or with alleles containing the *r*_15_ mutation in the cytoplasmic domain (Figure 4). However, susceptibility to Cry1Ac was lower for cells transfected with alleles containing the *r*_15_ mutation (Figure 5), which suggests that this mutation affected post-binding events.

Similar to previous data supporting the cell signaling model based on results with transfected H5 cells from the lepidopteran *Trichoplusia ni* [27], some of the data reported here indicating that the cytoplasmic domain of cadherin affects susceptibility to Cry1Ac are based on results with transfected Sf9 cells from the lepidopteran *Spodoptera frugiperda*. An important limitation of such data is that responses of cell cultures do not necessarily reflect responses of live insects. In particular, whereas the results from toxicity assays with Sf9 cells and live insects were qualitatively similar here, the increase in the LC_50_ value of Cry1Ac associated with *r*_15_ was greater for *H. armigera* larvae (82 to 140-fold, Table 1) than for transfected cells (2-fold, Figure 5). Nonetheless, the data reported here showing linkage of a cytoplasmic domain mutation with resistance to Cry1Ac provide the first evidence that this region affects susceptibility of live insects to Cry1Ac. Together with extensive data supporting the pore formation model [19], the results here suggest that both the pore formation pathway and an intracellular pathway contribute to the efficacy of Bt toxins, as previously hypothesized [41]. Nonetheless, we cannot exclude the alternative hypothesis that the cytoplasmic domain mutation confers resistance by interfering with post-binding events in the pore formation pathway.

We note that the resistance associated with cadherin allele *r*_1_, which carries a premature stop codon in the extracellular domain, was higher than the resistance associated with cadherin allele *r*_15_ (Table 1), which has an in-frame deletion expected to omit 55 amino acids in the cytoplasmic domain (Figure 1). These results are consistent with the hypothesis that both pore formation and cell signaling pathways contribute to toxicity, because the *r*_1_ mutation disrupts both the extracellular and cytoplasmic domains, whereas the *r*_15_ mutation is expected to affect the cytoplasmic domain, but not the extracellular domain.

The pattern of cross-resistance was similar for strain XJ-r15 with the cytoplasmic domain mutation (Table S2) and strain SCD-r1 with the extracellular domain mutation [35]. In both cases, the resistance ratio reflecting cross-resistance was highest for Cry1Aa (27 for XJ-r15 and >41 for SCD-r1), intermediate for Cry1Ab (6.3 for XJ-r15 and 31 for SCD-r1), and lowest for Cry2A (1.4 for Cry2Ab for XJ-r15 and 1.2 for Cry2Aa for SCD-r1). The lack of significant cross-resistance to Cry2A toxins in both strains is consistent with the idea that cadherin is not a receptor for these toxins.

Whereas non-recessive resistance to Bt toxins has been reported previously in *H. armigera* [29]–[33], the *r*_15_ allele identified here is the first case in which the molecular genetic basis of non-recessive resistance has been characterized in detail. The *r*_15_ allele differs from the two alleles conferring non-recessive resistance to Cry1Ac previously reported in the field-selected population of *H. armigera* from Anyang [29]. One of these two non-recessive alleles is not linked with cadherin. The other non-recessive allele (*r*_12_) is linked with cadherin and has amino acid substitutions in the putative toxin-binding region but not premature stop codons, deletions or insertions [29]. The discovery of the *r*_15_ allele considered together with earlier reports [29], [37] indicates that the Anyang population had at least four different types of mutations conferring resistance to Cry1Ac: non-recessive resistance associated with disruption of the intracellular domain of cadherin (*r*_15_), non-recessive resistance linked with substitutions in the extracellular domain of cadherin (*r*_12_), non-recessive resistance not linked with cadherin (strain AY423), and recessive resistance associated with disruption of the extracellular domain of cadherin (*r*_1_, *r*_2_, *r*_3_ and *r*_9_).

The resistance conferred by the *r*_15_ allele differs from the most common type of insect resistance to Bt toxins, which is called “Mode 1” and entails recessive inheritance, high levels of resistance to one or more Cry1A toxins, little or no cross-resistance to Cry1C, and reduced binding of one or more Cry1A toxins [42]. Examples of Mode 1 resistance include disruption of the extracellular region of cadherin associated with recessive resistance and reduced binding of one or more Cry1A toxins in *H. virescens*, *P. gossypiella* and *H. armigera* [20]–[22], [26], [43]–[45]. Disruption of an ABC transporter protein also confers recessive resistance to Cry1Ac in *H. virescens* [45], *Plutella xylostella* and *Trichoplusia ni* [46] and to Cry1Ab in *Bombyx mori* [47]. The first three of these cases with ABC transporter mutations also involve reduced binding of Cry1Ac [45], [48], [49] and thus fit the Mode 1 pattern, but reduced binding of Cry1Ab was not associated with resistance to this toxin in *B. mori* [47].

We hypothesize that the mechanism of non-recessive resistance to Cry1Ac associated with the *r*_15_ allele of *HaCad* is as follows: In *r*_15_ *s* heterozygotes, Cry1Ac binds to the mutant cadherin protein encoded by the *r*_15_ allele (half of the total), but this binding does not lead to toxicity because the *r*_15_ mutation interferes with post-binding events. This binding also reduces the amount of toxin available to cause toxicity by binding with the susceptible cadherin (the other half). Thus, sensitivity to Cry1Ac is substantially lower for *r*_15_ *s* heterozygotes than susceptible homozygotes, which yields non-recessive resistance. By contrast, when reduced binding of toxin is the primary mechanism of resistance as with the *r*_1_ allele of *HaCad*, Cry1Ac does not bind to the mutant cadherin in *r*_1_ *s* heterozygotes. This increases the amount of toxin available to bind with the susceptible cadherin and ultimately to cause toxicity via post-binding events. Therefore, even though only half of the cadherin protein binds Cry1Ac in *r*_1_ *s* heterozygotes, their net sensitivity to Cry1Ac similar to that of susceptible homozygotes, yielding recessive resistance.

The non-recessive inheritance detected here and previously in field-selected populations of cotton bollworm from China may have important practical implications for resistance management [29]. Better understanding of non-recessive resistance could be especially useful because the refuge strategy, the approach most widely adopted approach for delaying insect resistance to Bt crops, works best against recessive resistance [4], [6], [12]. Refuges consist of host plants that do not produce Bt toxins and thus allow survival of susceptible pests that can mate with resistant pests emerging from nearby Bt crops. Refuges delay resistance most effectively if resistance is recessive, because mating between homozygous resistant and homozygous susceptible pests produces heterozygous progeny that are killed by the Bt crop. Conversely, if resistance is not recessive and some of the heterozygous progeny survive on the Bt crop, refuges are expected to be less effective and it may be necessary to increase their abundance or use alternative approaches to delay resistance [50]. In northern China, non-Bt host plants other than cotton accounted for about 90% of the cropping area planted to *H. armigera* host plants, which may be a key factor slowing resistance [51]. Previous results showed that most resistant individuals in field-selected populations of northern China had non-recessive alleles, which suggests that increased attention is needed to monitor and manage non-recessive resistance of cotton bollworm to Bt cotton [29]. Further work will be needed to determine the impact of the *r*_15_ allele on evolution of resistance in the field.

## Materials and Methods

### Insect strains

The susceptible SCD strain of *H. armigera* was started with insects from the Côte d'Ivoire (Ivory Coast), Africa over 30 years ago and was maintained in the laboratory without exposure to insecticides or Bt toxins [35]. The *r*_1_ allele of the cadherin gene (*HaCad*) (previously called *Ha_BtR* [29], [35]) was isolated from the resistant strain GYBT, which was started in August 2001 with 300 late instars collected from Bt cotton in Gaoyang County of Hebei Province of northern China and selected with Cry1Ac for 28 generations in the lab [22]. The SCD-r1 strain was established by introgressing the *r*_1_ allele from the GYBT strain into the SCD strain. The SCD-r1 strain was fixed for the *r*_1_ allele and its LC_50_ was more than 400 times higher than the LC_50_ of the nearly isogenic SCD strain that lacked the *r*_1_ allele [35]. Larvae were reared on an artificial diet and adults were maintained as described previously [17].

We used the F_1_ screen method to detect alleles conferring resistance to Cry1Ac in *H. armigera* collected from the field during 2009 [29] (Table S1). No permits were required because all collections were made in China under the auspices of the Chinese Ministry of Agriculture. As described previously [29], male moths for the F_1_ screen were collected from light traps at three sites in northern China where Bt cotton had been planted intensively (Anci, Hebei Province in June; Anyang, Henan Province in June; Xiajin, Shandong Province in August). All of these field-collected male moths were crossed individually to virgin female moths from the SCD-r1 strain that were homozygous for the *r*_1_ allele (*r*_1_ *r*_1_). In addition, we collected fourth instars surviving on Bt cotton plants from Xiajin in July. We reared these field-collected larvae to pupation in the laboratory on artificial diet without Bt toxin and allowed moths to emerge. Each resulting field-derived male or female moth was crossed individually with a virgin moth of the opposite sex from the SCD-r1 strain.

We screened the F_1_ offspring from each of 572 single-pair families (n = 48 second instars per family) with a discriminating concentration of activated Cry1Ac (1 µg toxin per cm^2^ diet) [29], [38]. We scored families with larval survival >30% as resistant [29] and reared some survivors from these families to the final instar. Using methods described below, some of the survivors were held at −80°C for cadherin genotyping while others were reared to adults to establish resistant strains.

### Bt toxins and bioassays

Activated Cry1Ac, Cry1Aa, and Cry1Ab were provided by Dr. Marianne P. Carey (Case Western Reserve University, USA). Cry2Ab protoxin was provided by the Institute of Plant Protection, Chinese Academy of Agricultural Sciences (CAAS), China.

We used a diet surface contamination bioassay [38]. To allow direct comparison with previous bioassay data assessing responses to Cry1A toxins for second instars of *H. armigera* from China, we used the same bioassay method for Cry1A toxins that was employed in previous studies [17], [29], [38]. To conserve our limited supply of Cry2Ab and to allow direct comparison with previous Cry2Ab bioassay data from China [17] and Australia [36], we used the method established in Australia for testing Cry2Ab against first instars of *H. armigera* [36], which requires less toxin than the method for Cry1A toxins. Toxin stock suspensions were diluted with a 0.01 M, pH 7.4, phosphate buffer solution (PBS). PBS was used as a control. Liquid artificial diet (900 µl) was dispensed into each well of a 24-well plate. After the diet cooled and solidified, 100 µl of Bt toxin solution was applied evenly to the diet surface in each well and allowed to air dry. For Cry1A toxins, one second instar that had been starved for 4 h was placed in each well of the plate. For Cry2Ab, one unfed neonate larva (<24 h old) was placed in each well. Forty-eight larvae were tested for each toxin concentration. Larvae were scored as dead if they were dead or weighed less than 5 mg after 5 days for Cry1A toxins or 7 days for Cry2Ab at 26±1°C, with a 16∶8 L∶D photoperiod and 60% RH.

### Identification and detection of a cadherin allele (*r*_15_) with a cytoplasmic domain deletion

We extracted total RNA from larval midguts of a subset of survivors from the F_1_ screen from each of 48 resistant single-pair families using the SV total RNA isolation system according to the manufacturer's instructions (Promega, Madison, WI). We performed reverse transcription with the Moloney murine leukemia virus reverse transcriptase (Promega). We used four primer pairs to amplify four overlapping fragments that completely cover the cDNA of *HaCad* [36]. We used agarose gels to extract PCR products of the expected size and we purified these products with the Wizard DNA purification system (Promega, WI, USA) and cloned using the pGEM-T Easy Vector System (Promega). All clones were sequenced by Invitrogen (Shanghai, China).

We prepared genomic DNA from individual larvae or adults using a genomic DNA extraction kit according to the manufacturer's instructions (Axygen Biosciences, Union, CA). We designed a forward primer (Cyto-F in Exon32) and a reverse primer (Cyto-R in Exon 33) to amplify the genomic DNA flanking the mutation site of *r*_15_ (the primer sequences in Table S3). The amplification reaction mixture (25 µl) contained 100 ng of genomic DNA, 1 µM of each primer, 150 µM of each deoxynucleoside triphosphate, 2 mM of MgCl_2_, 1 U of rTaq DNA polymerase (TaKaRa, Dalian, China), and 2.5 µl of 10× PCR buffer. The PCR amplification protocol included denaturation at 94°C for 3 min, followed by 30 cycles (94°C for 30 s, 57°C for 1 min, and 72°C for 2 min) and a final extension at 72°C for 10 min. PCR products were analyzed by 1.5% agarose gel electrophoresis and ethidium bromide staining.

We used the banding patterns of PCR products to determine the cadherin genotype for the XJ-r15 strain. Susceptible homozygotes (*ss*) had only one fragment of 556 bp, resistant homozygotes (*r*_15_ *r*_15_) had only one fragment of 2,014 bp, and heterozygotes (*r*_15_s) had two fragments (2,014 bp and 556 bp) (Figure S5).

### Establishing resistant strains (XJ-r15 and AY-r15) homozygous for *r*_15_

To establish the first resistant strain homozygous for *r*_15_ (XJ-r15), we started with the first single-pair F_1_ family in which *r*_15_ was identified (Figure S2). The parents of this family were a field-collected male moth from Xiajin (*r*_15_ *s*) and a female moth from SCD-r1 (*r*_1_ *r*_1_). The F_1_ progeny from this family that survived exposure to the diagnostic concentration of Cry1Ac were *r*_1_ *r*_15_. These survivors were crossed with susceptible SCD adults (*ss*) to produce F_2_ progeny consisting of a mixture of *r*_1_ *s* and *r*_15_ *s*. Single-pair matings were made among the F_2_ progeny to generate F_3_ progeny consisting of a mixture of three genotypes including *r*_15_ *s* (Figure S2). After >300 eggs were collected from each single-pair family, cadherin genotypes of the parents of the F_2_ single-pair families were determined by two rounds of diagnostic PCR. The first round was made with primers r1-F/r1-R to detect the *r*_1_ allele [36]. Next we discarded families in which one or both parents had an *r*_1_ allele. For the remaining F_2_ families, in which *r*_1_ was not detected in either parent, we checked for the *r*_15_ allele using primers Cyto-F/Cyto-R. From families in which both parents were *r*_15_ *s*, we screened F_3_ larvae at 2.5 µg Cry1Ac per cm^2^ diet (2.5 times the diagnostic concentration). Survivors of this screen were used to establish the XJ-r15 strain. We used diagnostic PCR (Figure S5) to verify that individuals of XJ-r15 were homozygous for *r*_15_ (n = 10, all were *r*_15_ *r*_15_). Starting with the single-pair F_1_ family in which *r*_15_ was identified from Anyang, we used parallel procedures to establish resistant strain AY-r15, which was also homozygous for *r*_15_.

### Evaluation of dominance, maternal effects, and sex linkage

To evaluate dominance, maternal effects, and sex linkage, we used bioassays (as described above) to determine responses to Cry1Ac of the F_1_ progeny between a susceptible strain (SCD) and each of three resistant strains: XJ-r15 and AY-r15 with resistance allele *r*_15_ and SCD-r1 with resistance allele *r*_1_. For each of the three resistant strains, the F_1_ progeny were generated with reciprocal crosses as follows: We crossed 30 virgin female moths from the resistant strain with 30 male moths of the SCD strain and vice versa for the reciprocal cross. We used bioassays to determine the responses to Cry1Ac of F_1_ larvae from each reciprocal cross.

### Interstrain complementation test for allelism

To test for allelism between the *r*_1_ cadherin resistance allele in strain SCD-r1 and any major resistance alleles in strains XJ-r15 and AY-r15, we crossed SCD-r1 with XJ-r15 and AY-r15 and tested the F_1_ progeny using bioassays with Cry1Ac as described above. If resistance is not completely dominant in two resistant strains crossed in an interstrain complementation test for allelism, the F_1_ progeny from the cross will be more resistant if the resistance alleles occur at the same locus in both strains than if they occur at different loci in each strain [6], [29], [52].

### Analysis of genetic linkage with the cadherin locus

We used a genetic linkage analysis to determine if resistance to Cry1Ac in the XJ-r15 strain was genetically linked with the cadherin locus (Figure 3). We crossed a male from XJ-r15 with a female from the susceptible strain SCD to produce a family of hybrid F_1_ offspring. Next, we crossed an F_1_ male with a female from SCD to produce an F_2_ backcross family. Using the bioassay method described above, we reared larvae from the backcross family for five days on diet with either 0 (control), 0.3 or 0.5 µg Cry1Ac per cm^2^ diet. After five days, we transferred survivors to untreated diet and reared them until they reached the final instar. From each survivor to the final instar, we obtained genomic DNA that was used to determine the cadherin genotype of each individual using diagnostic PCR (Figure S4).

### Construction of recombinant pFastBac vectors with four cadherin alleles

We constructed vectors with four *HaCad* alleles: *s*, from the susceptible strain SCD, *r*_15_ from resistant strain XJ-r15, and two chimeric alleles (Figure S3). The chimeric allele *s*/*r*_15_ had the cytoplasmic domain from *r*_15_ and the other components from *s*, while the complementary chimeric allele *r*_15_/*s* had the cytoplasmic domain from *s* and the other components from *r*_15_. Tables S3 and S4 provide the sequences of the six primers and PCR conditions we used to construct these four alleles.

We amplified the coding sequence of *HaCad* from susceptible strain SCD (*s*) and resistant strain XJ-r15 (*r*_15_) with high fidelity PrimeSTAR® DNA HS polymerase (TaKaRa, Dalian, China) using the primers HaCad-Not1-F and HaCad-Xba1-R, respectively. With these two primers, Not1 and Xba1 restriction sites were introduced to the amplified fragments for subsequent in-frame cloning.

Overlap extension PCR was used to construct the two chimeric alleles. The first PCR amplified the ectodomain of *HaCad* from SCD and XJ-r15 strain using the forward primer HaCad-Not1-F and the reverse primer TMR. The second PCR amplified the cytoplasmic domain of *HaCad* from SCD and XJ-r15 strain using the forward primer TMF and the reverse primer HaCad-Xba1-R. Using the mixture of the first PCR amplification products of *HaCad* from SCD (*ss*) and the second PCR product from XJ-r15 (*r*_15_ *r*_15_) as template, a chimeric cDNA (*s*/*r*_15_) was amplified with the primers HaCad-Not1-F and HaCad-Xba1-R. Analogously, a complementary cDNA (*r*_15_/*s*) was amplified using the mixture of the first PCR amplification products of *HaCad* from XJ-r15 (*r*_15_ *r*_15_) and the second PCR product from SCD (*ss*) as template.

The four resulting PCR products of the expected sizes were excised and purified using the Wizard SV Gel and PCR Clean-Up System (Promega), and the four genes were ligated into the expression vector pFastBac1 (Invitrogen, Carlsbad, CA). Each of the four pFastBac1-HaCad plasmids (*ss*, *r*_15_ *r*_15_, *s*/*r*_15_, and *r*_15_/*s*) was transfected into *E. coli* TOP10 and selected for ampicillin-resistant transformants. We confirmed the presence and correct orientation of the inserts in the plasmids by restriction analysis and sequencing.

### Generating the recombinant bacmids and creating recombinant baculovirus stocks

Each of the four pFastBac1-HaCad plasmids was purified and transfected into *E. coli* DH10Bac to make recombinant bacmids (Bac-to-Bac Baculovirus Expression System, Invitrogen). The recombinant bacmids were purified and used to transfect *Spodoptera frugiperda* (Sf9) cells according to the manufacturer's protocol (Invitrogen). Transfection was achieved using Cellfectin reagent according to the manufacturer's instructions (Invitrogen). Viral supernatants from the initial P1 transfected cultures were harvested five days after transfection. Virus titers were improved through serial infections as recommended by the manufacturer (Invitrogen). P3 viral stocks containing the highest viral titer (estimated by serial dilution infections) were stored at 4°C and used for further transfections.

### Transfection of Sf9 cells

Sf9 cells (ATCC 1711-CRL) were cultured at 27°C in Sf-900 II SFM medium supplement (Invitrogen) with 10% fetal bovine serum and 10 µg/ml gentamycin. The cells were maintained at a density of 1×10^7^ cells/ml and were subcultured every 3–4 days. For infections, log phase cells were seeded at a density of 2×10^6^ cells/ml and infected with P3 recombinant baculovirus stocks in low serum media containing 2% fetal bovine serum. Sf9 cells were infected with recombinant viruses for each of the four recombinant cadherin alleles in six-well plates containing glass coverslips. Control Sf9 cells were either infected with an empty bacmid or were not infected.

### Detection of cadherin expression and binding of Cry1Ac of Sf9 cells

Two days after infection, cells were harvested and washed twice with PBS (pH 7.4) and fixed in 4% parafomaldehyde solution for 30 min at room temperature (RT). Fixed cells were washed three times with PBS (pH 7.4) and then blocked with 1% BSA for 1 h at RT. After blocking, they were incubated with rabbit polyclonal anti-Cadherin IgG antibody (1∶100 dilution) for 1 h at RT. The anti-cadherin antibody was raised against the toxin-binding portion of HaCad [53].

For immunolocalization of Cry1Ac binding, after BSA blocking the coverslips were washed in PBS and incubated with 10 nM Cry1Ac toxin for 2 h at 25–28°C. Coverslips were then washed as described above and incubated with rabbit polyclonal anti-Cry1Ac antibody (1∶100 dilution) for 1 h at 25–28°C.

After 1 h, the cells were rinsed three times with PBS, followed by incubation with FITC-conjugated goat IgG secondary antibodies (Promega, Shanghai) at 1∶100 dilution for 1 h. The unbound conjugate was removed by washing with PBS. The coverslips were sealed and examined immediately in a LSM Zeiss laser scanning confocal microscope, using excitation at 488 nm and 20× objective with additional zooming. Image acquisition of the controls and data processing were performed under the same conditions.

### Toxicity of Cry1Ac to Sf9 cells

Sf9 cells were infected with recombinant viruses in six-well plates with a multiplicity of infection of three. Three days after infection, cells were harvested and washed twice in PBS and centrifuged for 5 min at 500 g at 4°C. Cells were re-suspended in PBS buffer and counted in a hemacytometer. One hundred microliter aliquots containing 5×10^6^ cells were incubated for 1 h with or without Cry1Ac toxin. All incubations were performed in sterile 96-well cell culture plates at 27°C with pipetting repeated every 10 min to maintain homogenization. After incubation, two samples of 10 µl were transferred separately from each well into two new wells and thoroughly mixed with 10 µl of Trypan Blue solution (0.4% in 1× PBS). The number of live (unstained) and dead (stained blue) cells were counted in a hemacytometer. About 10,000 cells were counted for each replicate of each treatment. The experiment was repeated with three independent transfections for each of the six types of Sf9 cells (four transfected with cadherin alleles and two controls).

### Data analysis

We analyzed mortality data from diet bioassays with larvae and cytoxicity assays with Sf9 cells using probit analysis (DPS software, [54]) to estimate the concentration of toxin causing 50% mortality (LC_50_), the 95% fiducial limits (FL) of each LC_50_ value, as well as the slope of each concentration-mortality lines and the standard error (SE) of the slope. We calculated the resistance ratio of each strain or group of F_1_ progeny as its LC_50_ divided by the LC_50_ of the susceptible SCD strain. We considered LC_50_ values significantly different if their 95% fiducial limits did not overlap. We adjusted for control mortality (range = 0 to 6%) to estimate LC_50_ values, but not in the bioassays at only the diagnostic concentration, which had lower control mortality (<5%).

We estimated dominance (*h*) as described previously [34] using two methods: direct estimation of *h* based on survival at the diagnostic concentration of Cry1Ac and via estimation of *D* [55] based on LC_50_ values of Cry1Ac. *D* ranges from −1 (completely recessive) to 1 (completely dominant) while *h* ranges from 0 (completely recessive) to 1 (completely dominant). For comparison, we converted *D* to the same scale as *h* as follows: *h* = (*D*+1)/2 [34].

To quantify the results of interstrain complementation tests for allelism, we used the index of commonality (C) [29], which measures the extent to which resistance alleles in two resistant strains (R1 and R2) share a common locus as:
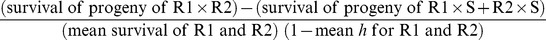
where S is a susceptible strain that has no resistance alleles and survival is measured at a single concentration, such as the diagnostic concentration. Values of C close to or <0 indicate the resistance alleles in the two strains do not share a common locus, while values of C close to or >1 indicate the two strains share a common locus. Note that C is most informative when *h* is close to zero for both strains, it becomes less informative as *h* approaches 1 for either resistant strain, and it cannot be calculated when *h* = 1 for both resistant strains. In this study, SCD was the susceptible strain (S), SCD-r1 was one resistant strain (R1), and either XJ-r15 or AY-r15 was the second resistant strain (R2). To calculate C, we used data from Table 1 on survival at the diagnostic concentration; for reciprocal crosses, we used the pooled data from the two crosses. For example, we calculated C for SCD-r1 and XJ-r15 as: (100−[0+40])/{([96+62]/2) (1−[0.65/2])} = 1.1.

## Supporting Information

Figure S1 **Genomic DNA sequence of exon 32 of** ***HaCad*** **in susceptible and resistant strains.** SCD: susceptible strain with wild type sequence. AY-r15: resistant strain with a 92 bp deletion. XJ-r15: resistant strain with a 1,459 bp insertion. Both mutations yield a predicted HaCad protein lacking 55 amino acids near the 5′-end of the cytoplasmic domain.
(TIFF)

Figure S2 **Marker-assisted selection used to produce resistant strains XJ-r15 and AY-r15, which are homozygous for the** ***r*** **_15_ allele.**
(TIFF)

Figure S3 **Cadherin in Sf9 cells transfected with four** ***HaCad*** **alleles.** As in Figure 1, the predicted protein structure includes an extracellular region (amino-terminal signal sequence [SIG], cadherin repeats [1]–[11], and membrane proximal region [MPR]), transmembrane region [TM], and cytoplasmic domain [C]. The four cadherin alleles are: susceptible (*s*), resistant (*r*_15_) causing a 55 amino acid deletion in C, chimeric allele *s*/*r*_15_ with C from *r*_15_ and the other components from *s*, and complementary chimeric allele *r*_15_/*s* with C from *s* and the other components from *r*_15_.
(TIFF)

Figure S4 **Detection of HaCad protein in Sf9 cells transfected with four alleles of** ***HaCad*** **by immunochemical analysis under confocal microscopy.** Cells were probed sequentially with anti-HaCad antiserum (1∶100) and FITC-conjugated anti-rabbit antibody (1∶100). HaCad was detected in Sf9 cells transfected with each of the four alleles of *HaCad*, but not in control Sf9 cells that were either transfected with an empty bacmid (EB) or not transfected. See Figure S3 for descriptions of the four cadherin alleles.
(TIFF)

Figure S5 **Diagnostic PCR for the** ***r*** **_15_ allele of** ***HaCad*** **in the XJ-r15 strain.** The primer pair Cyt-F/Cyt-R was used for PCR amplifications with genomic DNA as templates. The susceptible homozygote (*ss*) had one fragment of 556 bp, the resistant homozygote (*r*_15_ *r*_15_) had one fragment of 2014 bp, and the heterozygote (*r*_15_ *s*) had two fragments (2014 and 556 bp).
(TIFF)

Table S1 **Frequency of cadherin resistance allele** ***r*** **_15_ in F_1_ screens of three field populations of** ***H. armigera*** **sampled in northern China during 2009.**
(DOCX)

Table S2 **Cross-resistance of strain XJ-r15 of** ***H. armigera*** **, which had a resistance ratio of 140 against Cry1Ac relative to the susceptible strain SCD (see** **Table 1** **).**
(DOCX)

Table S3 **Primers used for amplifying the cadherin alleles of** ***H. armigera*** **.**
(DOCX)

Table S4 **PCR amplification protocols for recombinant** ***HaCad*** **alleles of** ***H. armigera*** **.**
(DOCX)
