# Supplementary figures and images for: Non-Recessive Bt Toxin Resistance Conferred by an Intracellular Cadherin Mutation in Field-Selected Populations of Cotton Bollworm
Source: PLoS One. 2012 Dec 28;7(12):e53418. doi: 10.1371/journal.pone.0053418 (PMC3532162; doi:10.1371/journal.pone.0053418)

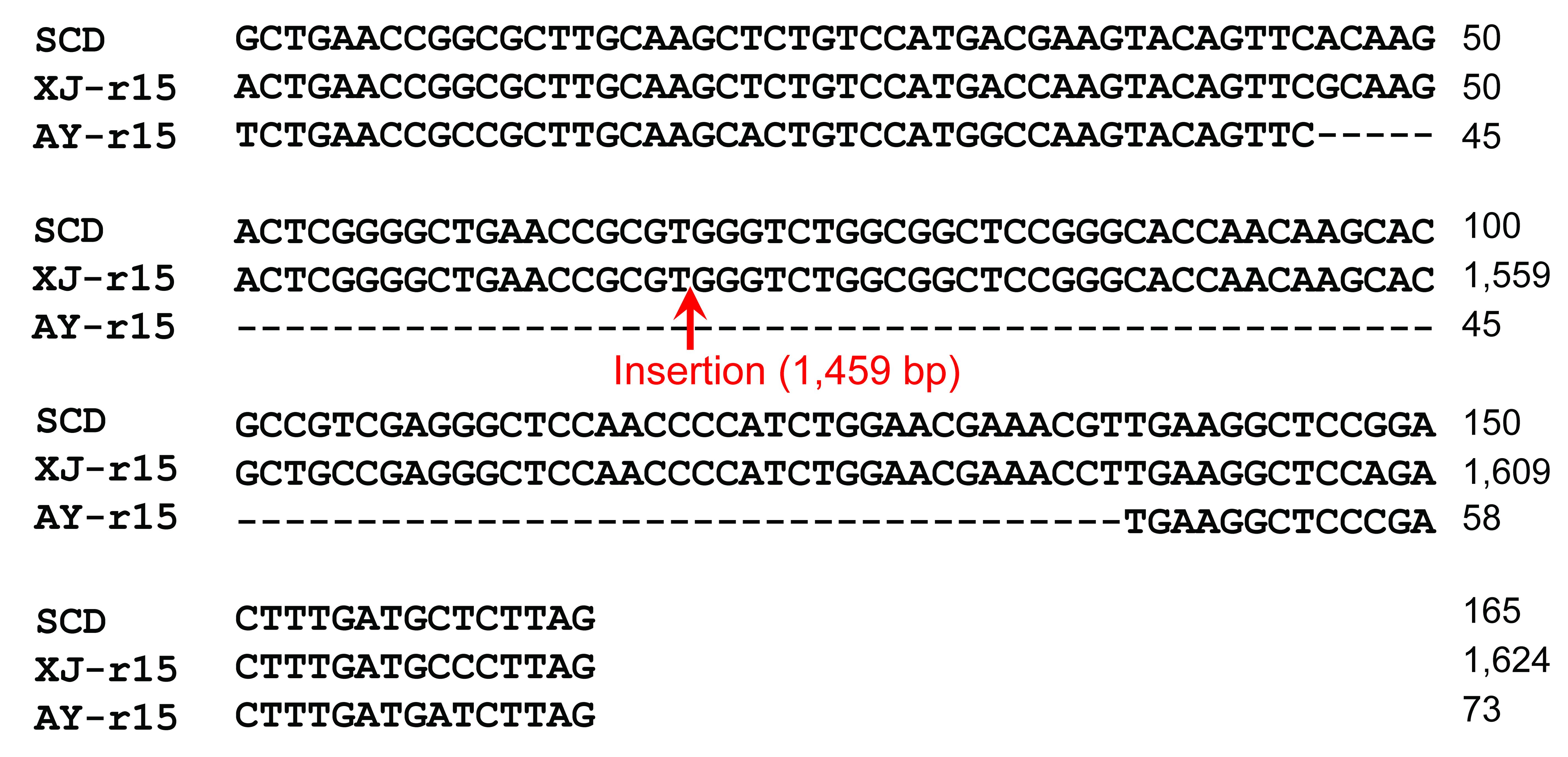

Supplement: Figure S1 — Genomic DNA sequence of exon 32 of HaCad in susceptible and resistant strains. SCD: susceptible strain with wild type sequence. AY-r15: resistant strain with a 92 bp deletion. XJ-r15: resistant strain with a 1,459 bp insertion. Both mutations yield a predicted HaCad protein lacking 55 amino acids near the 5′-end of the cytoplasmic domain. (TIFF) [file pone.0053418.s001.tiff]

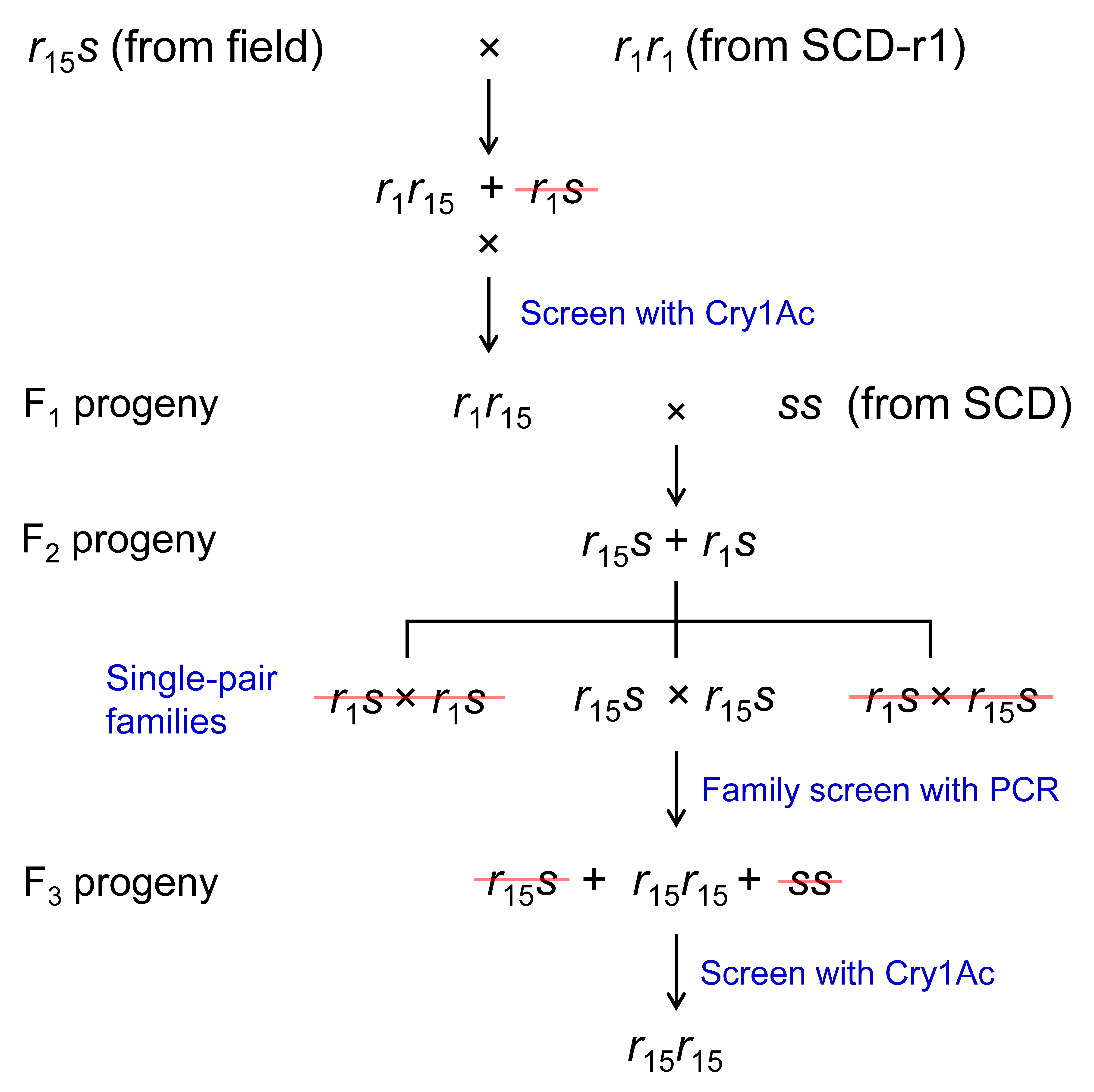

Supplement: Figure S2 — Marker-assisted selection used to produce resistant strains XJ-r15 and AY-r15, which are homozygous for the r 15 allele. (TIFF) [file pone.0053418.s002.tiff]

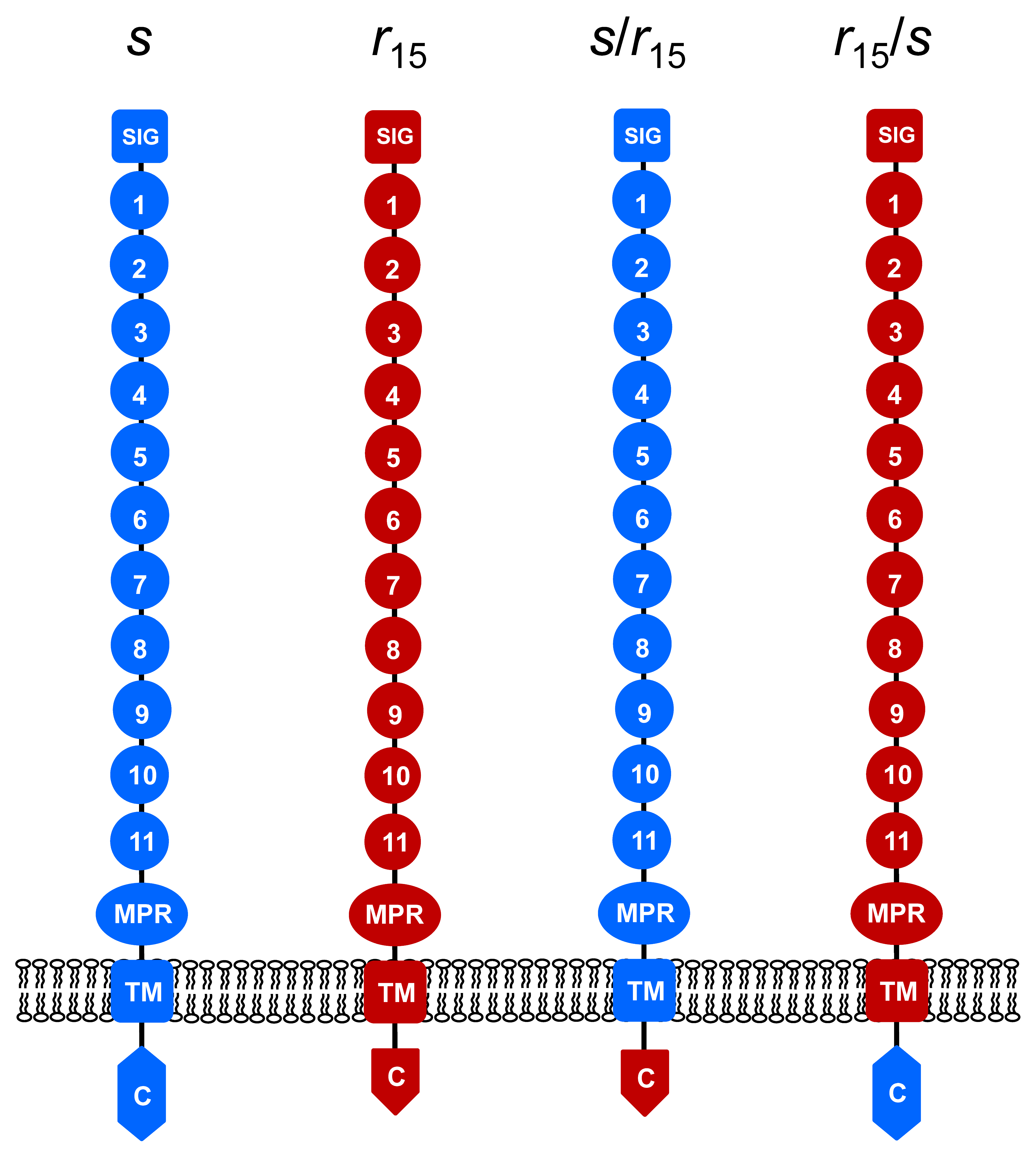

Supplement: Figure S3 — Cadherin in Sf9 cells transfected with four HaCad alleles. As in Figure 1, the predicted protein structure includes an extracellular region (amino-terminal signal sequence [SIG], cadherin repeats [1]–[11], and membrane proximal region [MPR]), transmembrane region [TM], and cytoplasmic domain [C]. The four cadherin alleles are: susceptible (s), resistant (r 15) causing a 55 amino acid deletion in C, chimeric allele s/r 15 with C from r 15 and the other components from s, and complementary chimeric allele r 15/s with C from s and the other components from r 15. (TIFF) [file pone.0053418.s003.tiff]

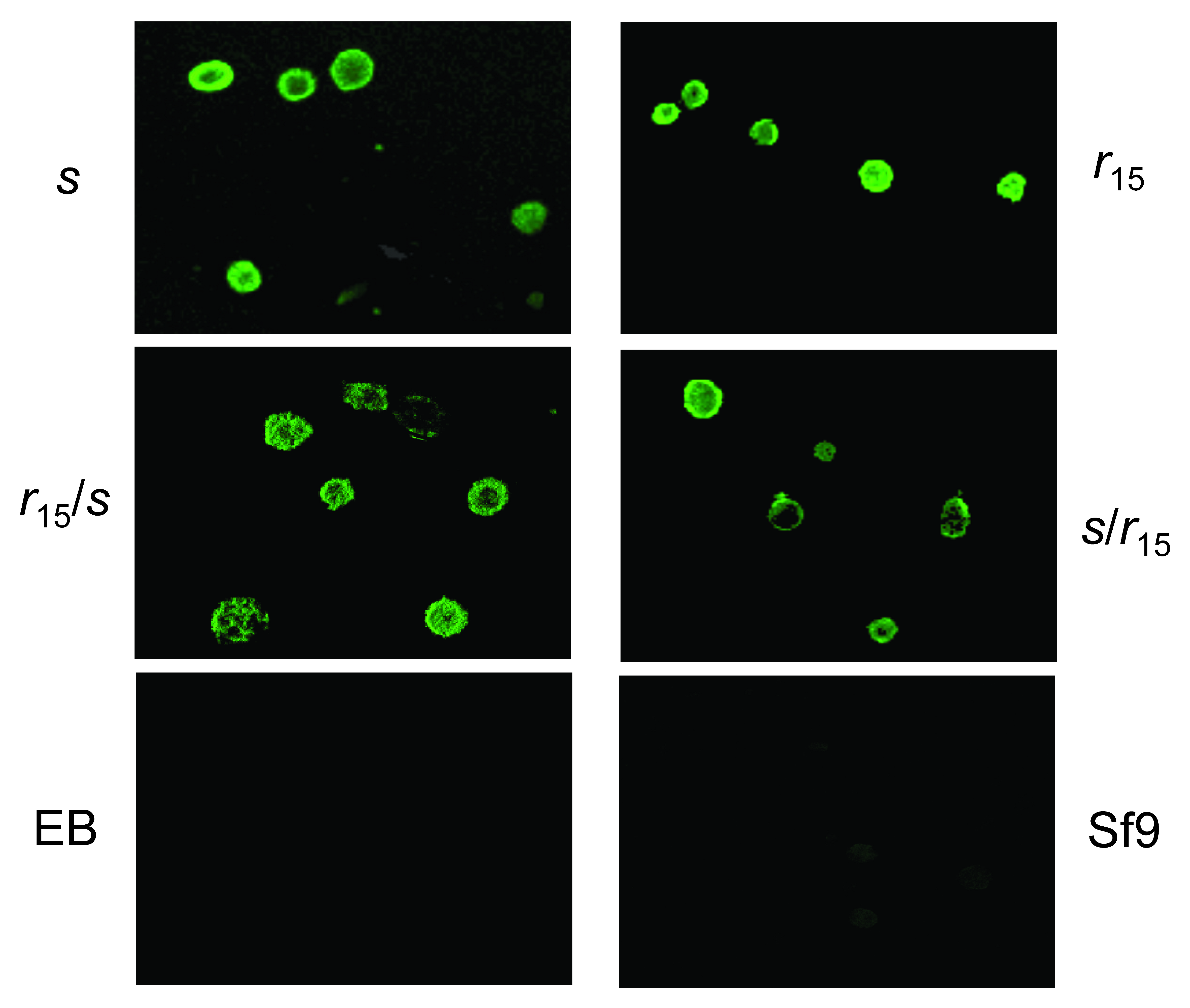

Supplement: Figure S4 — Detection of HaCad protein in Sf9 cells transfected with four alleles of HaCad by immunochemical analysis under confocal microscopy. Cells were probed sequentially with anti-HaCad antiserum (1∶100) and FITC-conjugated anti-rabbit antibody (1∶100). HaCad was detected in Sf9 cells transfected with each of the four alleles of HaCad, but not in control Sf9 cells that were either transfected with an empty bacmid (EB) or not transfected. See Figure S3 for descriptions of the four cadherin alleles. (TIFF) [file pone.0053418.s004.tiff]

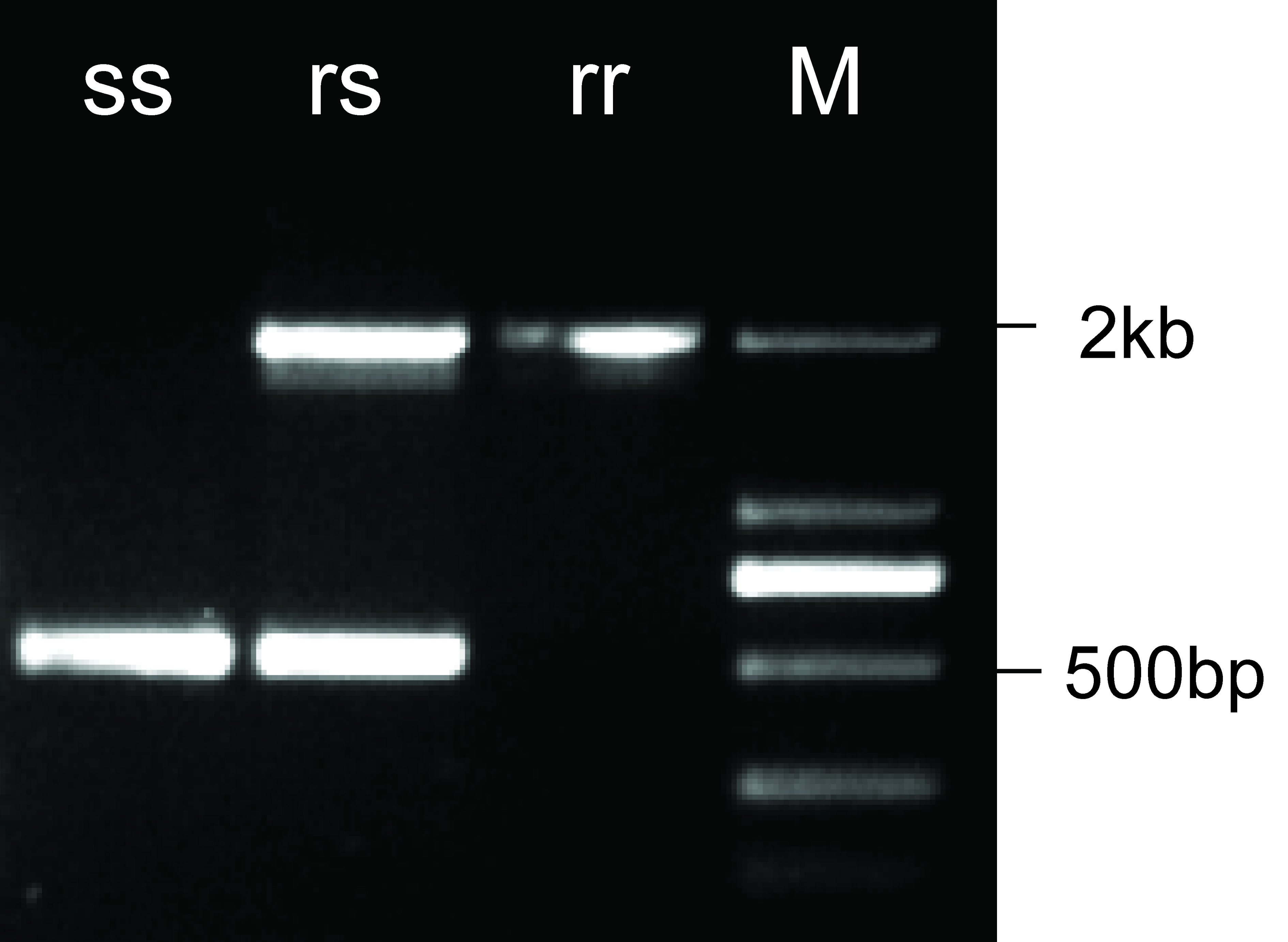

Supplement: Figure S5 — Diagnostic PCR for the r 15 allele of HaCad in the XJ-r15 strain. The primer pair Cyt-F/Cyt-R was used for PCR amplifications with genomic DNA as templates. The susceptible homozygote (ss) had one fragment of 556 bp, the resistant homozygote (r 15 r 15) had one fragment of 2014 bp, and the heterozygote (r 15 s) had two fragments (2014 and 556 bp). (TIFF) [file pone.0053418.s005.tiff]
