# Supplementary material for: Non-Recessive Bt Toxin Resistance Conferred by an Intracellular Cadherin Mutation in Field-Selected Populations of Cotton Bollworm
Source: PLoS One. 2012 Dec 28;7(12):e53418. doi: 10.1371/journal.pone.0053418 (PMC3532162; doi:10.1371/journal.pone.0053418)
Supplement: Table S1 — Frequency of cadherin resistance allele r 15 in F1 screens of three field populations of H. armigera sampled in northern China during 2009. (DOCX) [file pone.0053418.s006.docx]

**Table S1.** Frequency of cadherin resistance allele *r*_15_ in F_1_ screens of three field populations of *H. armigera* sampled in northern China during 2009.

| Population (Province) | Families  screened^a^ | Resistant  families^b^ | Resistant families sequenced^c^ | *r*_15_ alleles detected | *r*_15_ freq.^d^  (95% CI)^e^ |
| --- | --- | --- | --- | --- | --- |
| Xiajin (Shandong)^f^ | 230 | 67 | 20 | 1 | 0.0073  (0.0004 - 0.046) |
| Anyang (Henan) | 215 | 34 | 17 | 1 | 0.0047  (0.0003 - 0.030) |
| Anci (Hebei) | 127 | 11 | 11 | 1 | 0.0039  (0.0002 - 0.025) |
| Total | 572 | 112 | 48 | 3 | 0.0061  (0.0016 - 0.019) |

^a^ See Methods for details. Briefly, each single-pair F_1_ family was created by crossing a field-derived moth with a resistant moth from the SCD-r1 strain (*r_1_r_1_*). Larvae were exposed to a diagnostic concentration of Cry1Ac (1 μg Cry1Ac per cm^2^ diet) and families with larval survival >30% were scored as resistant [29].

^b^ The results for the number of resistant families were reported previously for all families from Anyang (215) and Anci (127) and for 146 of the 230 families from Xiajin [29]. We started the 84 additional families from Xiajin by collecting fourth instars surviving on Bt cotton. We started all other families by collecting moths as described previously [29].

^c^Number of resistant families in which cadherin cDNA was sequenced

^d^Frequency of *r*_15_ alleles = *r*_15_ alleles detected multiplied by (resistant families/families in which cadherin cDNA was sequenced) divided by (resistant field-derived individuals screened X 2 alleles per individual). For the total, this is 3 X (112/48) divided by (572 X 2) = 0.0061.

^e^We calculated the 95% confidence interval by the Wilson method with continuity correction using http://faculty.vassar.edu/lowry/prop1.html [29].

^f^ For Xiajin, one *r*_15_ allele was detected in the 84 families started with fourth instars surviving on Bt cotton (frequency = 0.021, 95% confidence interval = 0.0011 - 0.13), whereas no *r*_15_ alleles were detected in the 146 families started with male moths caught in light traps (frequency = 0, 95% confidence interval = 0.00 - 0.050).
