# Supplementary material for: Non-Recessive Bt Toxin Resistance Conferred by an Intracellular Cadherin Mutation in Field-Selected Populations of Cotton Bollworm
Source: PLoS One. 2012 Dec 28;7(12):e53418. doi: 10.1371/journal.pone.0053418 (PMC3532162; doi:10.1371/journal.pone.0053418)
Supplement: Table S2 — Cross-resistance of strain XJ-r15 of H. armigera , which had a resistance ratio of 140 against Cry1Ac relative to the susceptible strain SCD (see Table 1 ). (DOCX) [file pone.0053418.s007.docx]

**Table S2.** Cross-resistance of strain XJ-r15 of *H. armigera*, which had a resistance ratio of 140 against Cry1Ac relative to the susceptible strain SCD (see Table 1).

| Strain | Bt toxin | LC_50_ (95% FL)^a^ | Slope ± SE | n | RR^b^ |
| --- | --- | --- | --- | --- | --- |
| XJ-r15 | Cry1Aa | 6.0 (3.5-18) | 1.1 ± 0.3 | 240 | 27 |
|  | Cry1Ab | 1.7 (1.2-2.3) | 1.5 ± 0.2 | 240 | 6.3 |
|  | Cry2Ab | 0.075 (0.036-0.12) | 1.5 ± 0.2 | 288 | 1.4 |
| SCD | Cry1Aa | 0.22 (0.16-0.31) | 2.2 ± 0.2 | 288 | 1.0 |
|  | Cry1Ab | 0.27 (0.15-0.39) | 2.8 ± 0.4 | 288 | 1.0 |
|  | Cry2Ab | 0.055 (0.032-0.082) | 2.2 ± 0.2 | 288 | 1.0 |

^a^ Concentration killing 50% of larvae and 95% fiducial limits (µg toxin per cm^2^ diet).

^b^ Resistance ratio = LC_50_ of a strain divided by LC_50_ of the susceptible SCD strain.
