# Supplementary material for: Non-Recessive Bt Toxin Resistance Conferred by an Intracellular Cadherin Mutation in Field-Selected Populations of Cotton Bollworm
Source: PLoS One. 2012 Dec 28;7(12):e53418. doi: 10.1371/journal.pone.0053418 (PMC3532162; doi:10.1371/journal.pone.0053418)
Supplement: Table S3 — Primers used for amplifying the cadherin alleles of H. armigera . (DOCX) [file pone.0053418.s008.docx]

**Table S3.** Primers used for amplifying the cadherin alleles of *H. armigera*

| Primer name | Primer Sequence ( 5′＞3′) |
| --- | --- |
| Cyto-F | AAGTACAGTTCGCAAGACTCGG |
| Cyto-R | CGATCAGGTCTGAGTCGTATGAC |
| HaCad-Not1-F | ATAAGAATGCGGCCGCATGGCAGTCGACGTGAGAATACTGACG |
| HaCad-Xba1-R | TGCTCTAGATTATCTTCTGAACTGTGTGTTCGCG |
| TMF | GTTGTGCTGCTTATTGTGTTCTTTGTTAGGACTAGGAC |
| TMR | GTCCTAGTCCTAACAAAGAACACAATAAGCAGCACAAC |
