# Supplementary material for: Non-Recessive Bt Toxin Resistance Conferred by an Intracellular Cadherin Mutation in Field-Selected Populations of Cotton Bollworm
Source: PLoS One. 2012 Dec 28;7(12):e53418. doi: 10.1371/journal.pone.0053418 (PMC3532162; doi:10.1371/journal.pone.0053418)
Supplement: Table S4 — PCR amplification protocols for recombinant HaCad alleles of H. armigera . (DOCX) [file pone.0053418.s009.docx]

**Table S4.** PCR amplification protocols for recombinant *HaCad* alleles of *H. armigera*

| PCR product | Primer combination | PCR cycling program | PCR reaction mixture |
| --- | --- | --- | --- |
| Ectodomain | Forward: HaCad-Not1-F  Reverse: TMR | 30 cycles of 10 sec at 98^o^C,  5 sec at 59^o^C, 6 min at 72^o^C. | 25 μl reactions containing 200 ng of cDNA, 0.2 μM of each primer, 200 μM of dNTPs, 2.5 mM of MgCl2, 1 U of LA Taq DNA polymerase (with proofreading activity), and 2.5 μl of 10× LA PCR buffer. |
| Cytodomain | Forward: TMF  Reverse: HaCad-Xba1-R | 30 cycles of 10 sec at 98^o^C,  5 sec at 61^o^C, 45 sec at 72^o^C. |  |
| Full length | Forward: HaCad-Not1-F  Reverse: HaCad-Xba1-R | 30 cycles of 10 sec at 98^o^C,  5 sec at 59^o^C, 6 min at 72^o^C, and a final extension at 72^o^C for 10 min. |  |
